# 17β-Estradiol promotes LC3B-associated phagocytosis in trained immunity of female mice against sepsis

**DOI:** 10.7150/ijbs.53050

**Published:** 2021-01-01

**Authors:** Zhiheng Sun, Junxing Qu, Xiaoyu Xia, Yuchen Pan, Xinghan Liu, Huaping Liang, Huan Dou, Yayi Hou

**Affiliations:** 1 The State Key Laboratory of Pharmaceutical Biotechnology, Division of Immunology, Medical School, Nanjing University, Nanjing, China.; 2 State Key Laboratory of Trauma, Burns and Combined Injury, Department of Wound Infection and Drug, Daping Hospital, Army Medical University, Chongqing, China.; 3 Jiangsu Key Laboratory of Molecular Medicine, Division of Immunology, Medical School, Nanjing University, Nanjing, China.

**Keywords:** estradiol, trained immunity, sepsis, LAP, RUBICON, ROS

## Abstract

Sepsis is a common serious clinical infectious disease accompanied by more severe injuries and higher mortality rates in men than women. The much higher level of 17β-estradiol (E_2_) in female is one of the significant reasons for better sepsis resistance ability. Trained immunity is a novel way to fight against infection by improving innate immunity. However, whether β-glucan-induced trained immunity can promote macrophage phagocytosis to clear infections in early sepsis has not been clarified. And whether E_2_ involved in this process needs further investigation. Symptoms among male, female and ovariectomized (OVX) C57BL/6 mice in early sepsis were detected. The effect of trained immunity on macrophage LC3B-associated phagocytosis (LAP) and the mechanism of E_2_ functioned in this process have also been explored. We demonstrated compared with male mice, female has significantly more mild symptoms and more reactive oxygen species (ROS) production and stronger NADPH oxidase 2 (NOX2) expression in the macrophage of major organs. In contrary, these characteristics are disappeared in OVX mice. Furthermore, in macrophage cell lines and primary bone marrow- derived macrophages (BMDMs), β-glucan-induced trained immunity can increase ROS production by activating NOX2 to promote macrophage LAP. E_2_ can up-regulate RUBICON through estrogen receptor α (ERα) to further facilitate macrophage LAP. These results indicated that trained immunity can improve sepsis resistance ability by stimulating macrophage LAP. E_2_ can boost ROS production and RUBICON expression to further promote macrophage LAP, which can provide a new perspective to recognize the mechanism of trained immunity in gender differences when responding to sepsis.

## Introduction

Sepsis is a life-threatening organ dysfunction caused by a host's dysfunctional response to infection. Fungi, viruses, parasites and especially bacterial can all trigger sepsis 1. The occurrence of sepsis can lead to a very high mortality rate of 30% to 50%. Even with the development of modern medicine, the clinical mortality of sepsis has not been controlled at a low level 2. When the body is in sepsis state, the main organs will be damaged. Many key biomarkers of sepsis have been discovered and explored 3. Among them, damage of kidney, liver and lung are vital hallmark features and are accompanied by increased renal bacterial burden, higher transaminase and lactic acid levels in serum.

Mice sepsis model is commonly established by cecal ligation and puncture (CLP) or direct intraperitoneal injection of *E. coli* or lipopolysaccharide (LPS, a substance present on the cell wall of Gram-negative bacteria). Notably, some researches have shown that sepsis has a significant sexual dimorphism which indicates females have much lower severity and mortality after infection induced sepsis 4, 5. Based on these phenomena, some articles attribute the gender differences in sepsis to different hormone levels between males and females 6-8. Hormones in females are mainly divided into two categories: estrogen and progesterone. Among them, as the most predominant and potent endogenous estrogen 9, it is of great clinical significance to investigate the mechanism of 17β-estradiol (E_2_) against sepsis in females. Also, estrogen receptor is the first nuclear receptor discovered in 1950s by Elwood V. Jensen 10. However, to date, it is unclear how E_2_ regulates transcriptional genes through estrogen receptors and thereby regulates trained immunity. The mechanism may provide a possible way to improve the treatment of sepsis in both male and female.

The immune system can be divided into non-specific innate immunity and specific acquired immunity with “immune memory”. Nevertheless, the discovering of trained immunity in 2011 broke this traditional perception at a certain level 11, 12. Trained immunity is a novel type immunity that occurs in innate immune cells, especially macrophages. When macrophages are stimulated by antigens (such as β-glucan) for the first time, a series of cell biological changes will occur, including enhanced glycolysis, epigenetic changes, and activation of Akt-mTOR signaling pathway 13. Thereafter, when encountering the second antigen challenge (not necessarily the same as the first time antigen type), the macrophages will produce a faster and stronger immunological response, thus helps host to resist a wider types of subsequent infections 13, 14. The above-mentioned phenomenon suggests that trained immunity enables innate immune cells to acquire “immune memory” similar to acquired immunity, and can produce more timely and intensive response to infections against a wider range of subsequent infections 15. In the resistance process of bacterial infection-induced sepsis, trained immunity offers a creative solution for sepsis treatment ideas and methods. However, the current research on macrophage trained immunity mainly focuses on the promotion of inflammatory cytokines secretion, few research has focused on whether trained immunity can facilitate pathogenic bacteria elimination ability by promoting macrophage phagocytosis. And whether E_2_ participate in this process needs further investigation.

Macrophages can produce both NOX2 and ROS, which play a vital role in killing pathogens and fighting infections. Clinically, the lack of NOX2 function can lead to chronic granulomatous disease (CGD) 16. When pathogens bind to cell surface immune receptors TLR and FcγR during phagocytosis, LC3 can be recruited to macrophage phagosomes, thereby promoting phagocytosis 17. However, this process can be inhibited by NOX inhibitor Diphenyleneiodonium chloride (DPI) and NOX2 depletion. The inhibitory effect of N-acetyl-L-cysteine (NAC) is not obvious 18. Due to the recruitment of LC3 to phagosome surface, this process is called LC3B-associated phagocytosis (LAP). Compared to classical autophagy which occurs when cells are deprived of nutrition or growth factors, LAP is a non-canonical autophagy. There are similarities and differences between classical autophagy and LAP. Both of them need LC3B recruite to phagosome and share same proteins, most of which are ATG family proteins 19. However, phagosome in LAP is not double-layer membrane structure but single-layer. LAP starts from phagocytosis but not endoplasmic reticulum 20. Some studies showed that LAP is one of major methods of pathogen elimination in macrophage. Macrophages recognize pathogens through receptors to form phagosome and ROS are generated by NADPH oxidase to finish pathogenic bacteria killing and elimination. The initiation of LAP requires activation of NADPH oxidase and RUBICON 21. As the main NADPH oxidase in phagocytes, NOX2 is composed of membrane-bound gp91^phox^ (Cybb), p22^phox^ and cytoplasm p67^phox^, p47^phox^, p40^phox^ and small GTPase Rac1/2 22. There are six subunits that can transfer electrons from cytoplasmic NADPH to oxygen in phagosome to produce ROS. As a main mediator, RUBICON can inhibit autophagy but facilitate LAP 23. RUBICON can also stabilize NOX2 complex to maintain ROS production to ensure bacteria removal effect of LAP 24. However, the regulatory effects of trained immunity on NOX2 and RUBICON, as well as macrophage LAP process are unclarified. The investigation of these effects can help us understand the cause and mechanism of trained immunity from a new perspective.

Here, we demonstrate that β-glucan-induced trained immunity can assist female mice to fight sepsis better than males. When it comes to OVX mice, the effect of trained immunity is decreased so as to lead to the advantage disappearance in resisting sepsis. Meanwhile, β-glucan-induced trained immunity can upregulate expression of RUBICON and NOX2 in macrophages, and facilitate ROS production, thereby promoting LAP process to enhance pathogen engulf ability. In addition, *in vitro* experiments showed that E_2_ can further promote trained immunity to facilitate LAP. Our results indicated that higher levels of E_2_ in females can increase macrophage LAP to eliminate pathogens, thereby making females more resistant to sepsis. These may explain the reasons of sepsis gender dimorphism from an angle.

## Results

### β-glucan-induced trained immunity allows females to better resist sepsis and higher levels of ROS and NOX2 in main organs than males

**Figure 1:**
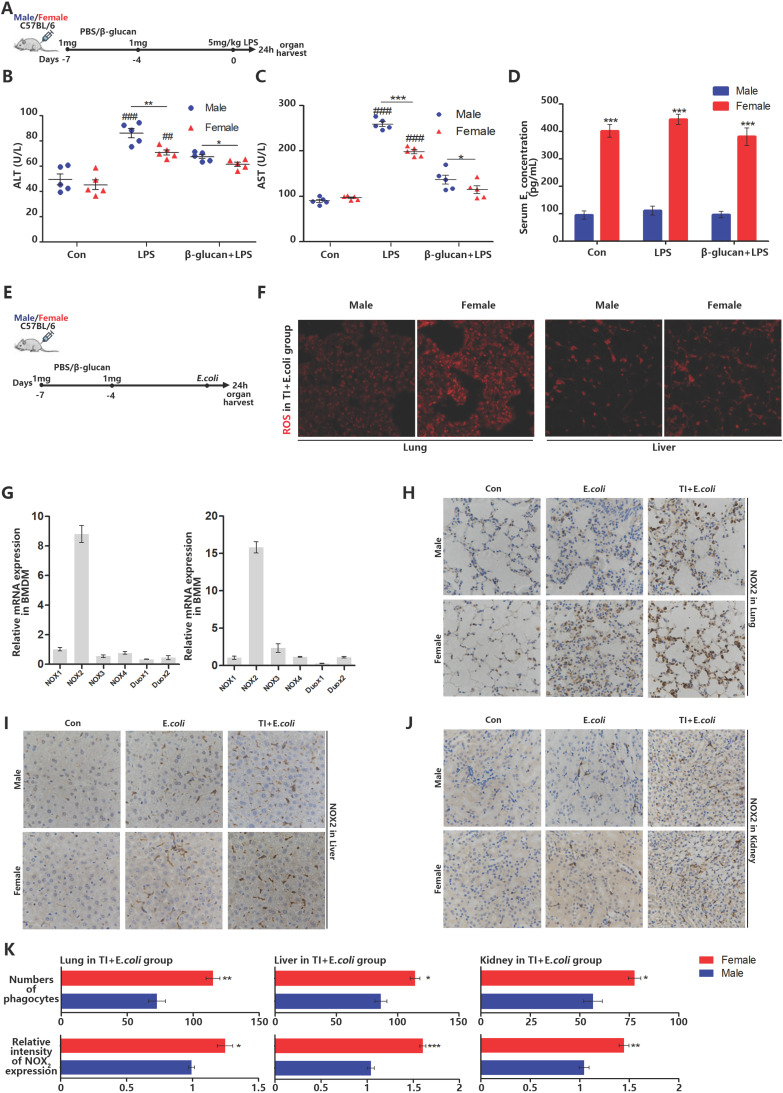
** β-glucan-induced trained immunity allows females to better resist sepsis and higher levels of ROS and NOX2 in main organs than males.** (A) *In vivo* model of trained immunity (TI) by two intraperitoneal (i.p.) β-glucan injections and secondary i.p. LPS challenge (n=5/group). (B and C) Levels of serum ALT and AST were detected in female and male mice treated with LPS or TI + LPS groups. (D) Serum concentration of estradiol (E_2_) was analyzed by ELISA in LPS and TI + LPS groups. Each panel is a representative experiment at least three independent biological replicates. (E) *In vivo* trained immunity model was established by two intraperitoneal (i.p.) β-glucan stimulations and secondary i.p. *E.coli* challenge (n=5/group). (F) ROS staining in lungs and livers of male and female mice in TI + *E.coli* group. (G) mRNA expression of various NADPH oxidases in mouse bone marrow derived macrophage (BMDM) and bone marrow derived monocyte (BMM). The monocyte-macrophage system is NOX2. (H-J) Immunohistochemical lung, liver and kidney NOX2 staining of male and female mice in the control group, TI group and TI + *E.coli* group. (K) Comparison of the number of phagocytes and their NOX2 expression level in lung, liver and kidney between male and female mice in TI +* E.coli* group. As mice phagocytes specifically express NOX2. *p<0.05, **p<0.01 and ***p<0.001 comparing female and male. In (B)-(C), single dots correspond to individual mice, *p<0.05, **p<0.01 and ***p<0.001 comparing in the same experimental group. ##*p<*0.01 and ###*p<*0.001 comparing control group, LPS group and TI + LPS group in same gender. In (D), ***p<0.001 comparing in the same experimental group between male and female.

Sepsis can be regarded as an infectious disease in the early stage. As the disease progresses, sepsis can over-activate immune system to produce a cytokine storm, attacks normal tissues and organs, resulted in tissue damage even death 25. Therefore, sepsis is an infection disease in the early stage but an autoimmune disease in the later stage. In the investigation of sepsis, it was found that females are better than males in both the incidence of sepsis and the mortality. Trained immunity can enhance the expression of inflammatory cytokines in innate immune cells. Our previous article showed that in the process of fighting early sepsis infection, trained immunity can promote macrophage M1 polarization 26. However, macrophages can also fight infection by recognizing and engulfing pathogens 27. In order to explore the effect of trained immunity on phagocytic ability in immune system, β-glucan-induced trained immunity and LPS-induced sepsis model was established in female and male mice (Figure 1A). Firstly, liver damage markers, aspartate aminotransferase (AST) and alanine aminotransferase (ALT), were significantly higher in males than females. After trained immunity, both males and females have significantly reduced liver damage but females are better than males (Figure 1B and 1C). Furthermore, the serum estradiol level of female in three groups was much higher than that of males (Figure 1D). When body cells especially macrophages fight infection, they produce ROS to kill invading pathogens. The amount of ROS not only reflects the immune intensity against infection, but also ROS and its producer (NADPH oxidases) are vital mediators that can regulate macrophage phagocytic signaling pathway 28. Once lack of ROS production, even if macrophages engulfed pathogens but without ability to kill them, will lead to lethal chronic granulomatous disease (CGD). Thus, to better simulate bacterial infection, we also set up TI and sepsis model by i.p. injection of β-glucan and *E.coli* (Figure 1E). Female lungs and livers produce more ROS than males when they fight against sepsis after trained immunity (Figure 1F). There are many types of NADPH oxidases, and each tissue cells specifically expresses its certain type of NADPH oxidase 29. Phagocytes specifically express NOX2 and our data indicated that both monocytes and macrophages derived from mouse bone marrow also specifically express NOX2 (Figure 1G). Since the monocyte-phagocytic cell system in mouse organs mainly includes monocytes, macrophages and dendritic cells and the majority of them are macrophages 30. Thus, NOX2 positive cells are mainly macrophages 31. By immunohistochemical NOX2 staining of lung, liver and kidney, it was found that female mice contained more phagocytes with higher NOX2 expression than males in *E.coli* and TI + *E.coli* group (Figure 1H-1K). These results suggested that females express more NOX2 in trained immunity and thus produce more ROS to acquire better sepsis resistance than males.

### Trained immunity manifests serious sepsis consequences and the decreased ROS and NOX2 levels in OVX mice than female mice

**Figure 2:**
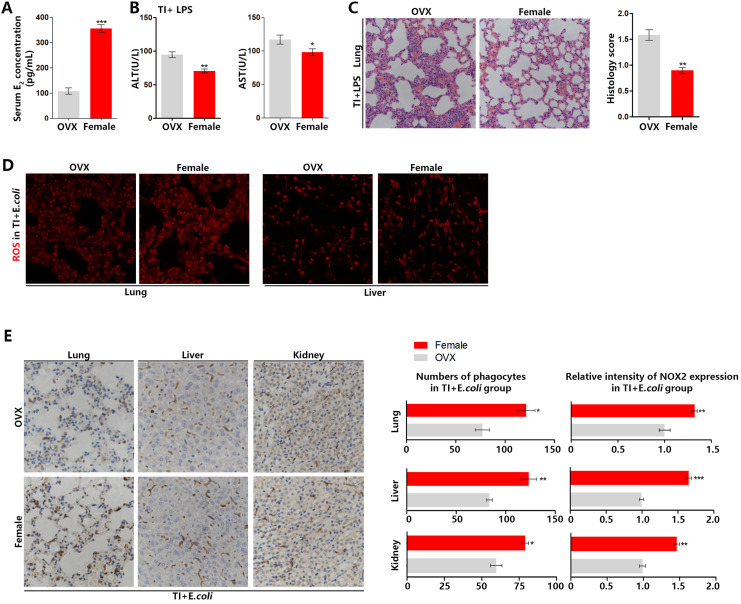
** Trained immunity manifests serious sepsis consequences and decreased ROS and NOX2 levels in OVX mice than female mice.** (A) Serum E_2_ concentration was significantly decreased in OVX mice. (B) Levels of serum ALT and AST were detected in OVX and female mice in TI + LPS group. (C) Lung was visualized by H&E staining. And microscopically analyzed and histologically scored by a pathologist. Trained immunity model of (A)-(C) was established according to Figure 1A. (D) ROS staining in lung and liver of OVX and female mice in TI + *E.coli* group. (E) Immunohistochemical lung, liver and kidney NOX2 staining of OVX and female mice in TI + *E.coli* group (n≥5/group). Trained immunity model of (D)-(E) was set up according to Figure 1E. *p<0.05, **p<0.01 and ***p<0.001 comparing OVX and female mice.

Ovary is the main organ that produces estrogen. To confirm the difference between male and female mice during fighting sepsis after trained immunity is caused by estrogen, we established trained immunity and sepsis model in OVX mice. In Figure 2A-2C, the model was established as Figure 1A. The serum E_2_ level of OVX mice is much lower than that of females (Figure 2A). In resisting sepsis after trained immunity, OVX mice have more severe liver damage than female mice (Figure 2B). The H&E histochemical staining also showed that OVX mice lung injury was more severe (Figure 2C). In addition, by setting up TI and sepsis model according to Figure 1E, in frozen sections of lung and liver, OVX mice also produced less ROS than females (Figure 2D). By performing NOX2 staining on the immunohistochemical sections of lung, liver and kidney, the results showed that the number of phagocytes and NOX2 expression in the main organs of OVX mice were significantly lower than those of females (Figure 2E). These demonstrated that estrogen can promote sepsis resistant ability of trained immunity by up-regulating the number of phagocytes (mainly macrophages) and ROS production in mouse main organs.

### Estradiol and trained immunity increase ROS level and promote phagocytic ability in macrophages

**Figure 3:**
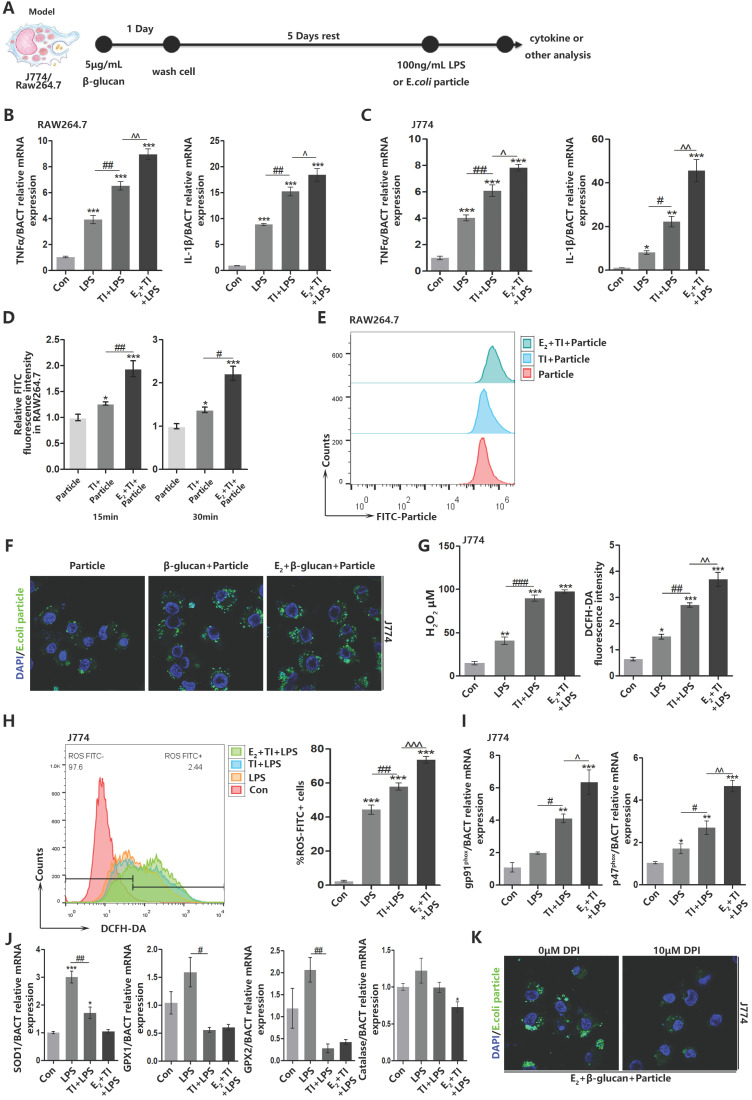
** Estradiol and trained immunity increase ROS level and promote phagocytic ability in macrophages.** (A)* In vitro* trained immunity model for J774 and RAW264.7 cell lines. (B) The mRNA levels of TNFα and IL-1β in RAW264.7 were detected by qPCR to determine trained immunity effect with or without E_2_. (C) The mRNA levels of TNFα and IL-1β in J774 were detected by qPCR to determine trained immunity effect with or without E_2_. (D) Fluorescence intensity detection of RAW264.7 phagocytosis of *E.coli* particles in 15 min and 30 min. (E) Flowcytometric detection of amount of *E.coli* FITC particles phagocytosed by macrophages at 15 min. (F) Immunofluorescence observation of the amount of *E.coli* particles engulfed by J774 at 15 min in particle, TI + particle and E_2_ + TI + particle groups. (G) The concentration of hydrogen peroxide and total amount of ROS of J774 in control, LPS, TI + LPS and E_2_ + TI + LPS groups. (H) Flowcytometric detection of total ROS of J774 in control, LPS, TI + LPS and E_2_ + TI + LPS groups. (I) The mRNA levels of gp91^phox^ and p47^phox^ (NOX2 subunits) of J774 were detected by qPCR in control, LPS, TI + LPS and E_2_ + TI + LPS groups. (J) The mRNA levels of ROS scavenging enzymes of J774 were detected by qPCR in control, LPS, TI + LPS and E_2_ + TI + LPS groups. (K) Immunofluorescence observation found when NOX2 inhibitor DPI is added, the phagocytic ability of J774 was decreased in E2 + TI + particle group (n≥3/group). *p<0.05, **p<0.01 and ***p<0.001 when compared to control or particle group. #*p<*0.05, ##*p<*0.01 and ###*p<*0.001 comparing LPS group and TI + LPS group or comparing TI + particle group and E_2_ + TI + particle group. ^*p<*0.05, ^*p<*0.01 and ^^^*p<*0.001 comparing TI + LPS group and E_2_ + TI + LPS group.

To explore the reason why estrogen can enhance ROS level in trained immunity macrophages, we established trained immunity and sepsis model in macrophage cell lines J774 and RAW264.7 (Figure 3A). Firstly, for β-glucan induced trained immunity can strengthen inflammatory cytokines TNFα and IL-6 expression, we confirmed that trained immunity model is successfully established and E_2_ can further facilitate trained immunity (Figure 3B and 3C). After trained immunity, RAW264.7 was accompanied by intracellular ROS enhancement at 15 and 30 min. At 15 min, the production of ROS in macrophages can already be upregulated (Figure 3D). The *E.coli* particles used in the experiment can have green fluorescence only in low pH environment. When the particles were engulfed, the phagosomes will fuse with lysosomes and turn to low pH environment. Detection of macrophage fluorescence intensity can verify phagocytic level. Flow cytometry found that E_2_ and trained immunity can increase RAW264.7 phagocytosis when *E.coli* particles are added for 15 min (Figure 3E). Similarly, immunofluorescence observation also found that E_2_ and trained immunity can promote J774 phagocytic ability (Figure 3F). Fluorescence and flow cytometry have shown enhancement of J774 phagocytic ability after trained immunity is accompanied by the production of intracellular hydrogen peroxide and total ROS (Figure 3G and 3H). NOX2 is the main source of ROS production in macrophages. As indispensable subunits of NOX2, gp91^phox^ and p47^phox^ play a vital role in keeping normal production of ROS by NOX2 and mediating signaling pathways in LAP. E_2_ and trained immunity can up-regulate mRNA expression of gp91^phox^ and p47^phox^ (Figure 3I). At the same time, many enzymes can eliminate excessive intracellular ROS levels 32, and E_2_ and trained immunity can reduce the mRNA levels of these enzymes (Figure 3J). When adding NOX2 inhibitor DPI to J774 in E2 + TI group, it will inhibit J774 phagocytic ability (Figure 3K). These results indicated E_2_ and trained immunity increase macrophage phagocytosis accompanied by an enhancement in ROS levels. E_2_ and trained immunity on the one hand can promote the NOX2 expression, on the other hand reduce ROS scavenging enzymes expression. Furthermore, inhibiting ROS production will in turn suppress macrophage phagocytosis.

### NOX2 inhibitor inhibits ROS levels in trained immunity of female mice and weakens sepsis resistant ability

**Figure 4:**
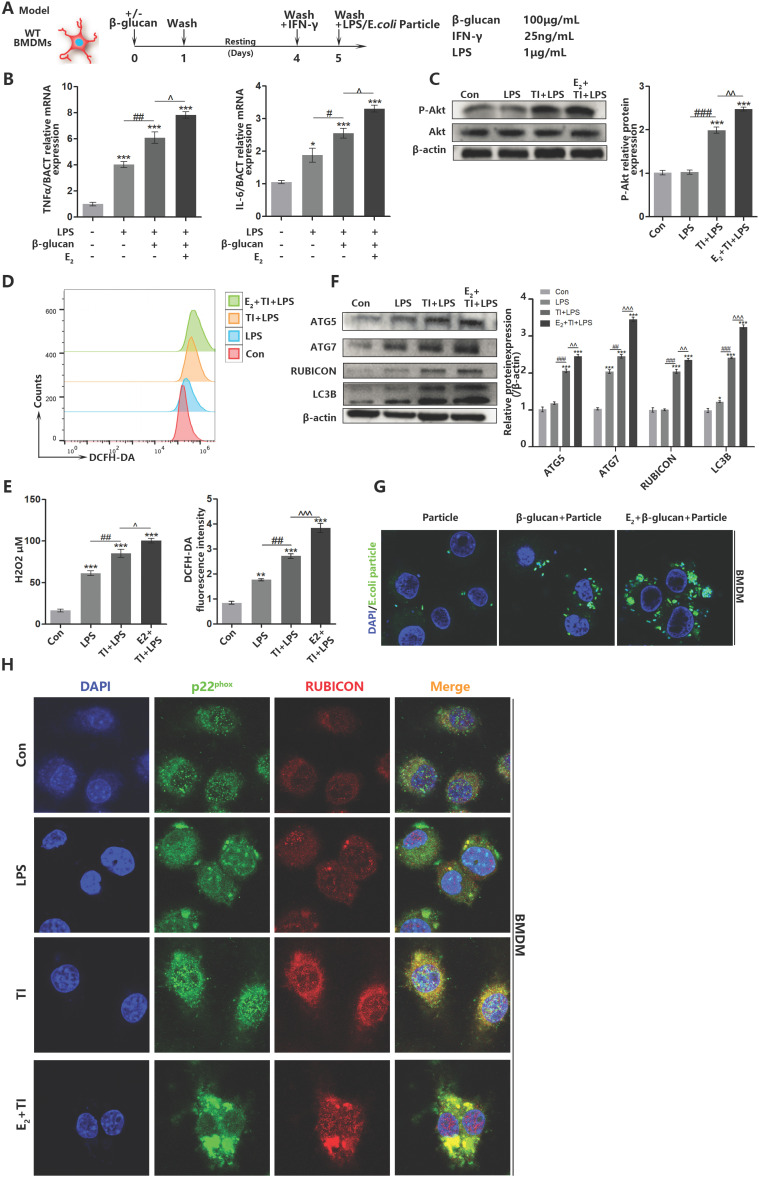
** NOX2 inhibitor inhibits ROS levels in trained immunity of female mice and weakens sepsis resistant ability.** (A) Inhibition of ROS model with DPI in female trained immunity mice *in vivo*. (B) Lung was visualized by H&E staining. And microscopically analyzed and histologically scored by a pathologist. (C) Levels of serum ALT and AST were detected in DPI and non-DPI group. (D and E) ROS staining in lung and liver of DPI and non-DPI in female TI + *E.coli* group. (F) Serum IL-6 and TNFα were measured by ELISA to characterize the effect of ROS inhibition on systemic inflammatory response (n≥3/group). **p<0.01 and ***p<0.001 comparing DPI + TI + *E.coli* and TI + *E.coli* group of female mice.

To confirm the effect of ROS on sepsis resistant ability after trained immunity, we established a model of inhibiting ROS with NOX2 inhibitor DPI in trained immunity female mice (Figure 4A). After ROS inhibition, lung and liver injury in female mice with sepsis increased (Figure 4B and 4C). DPI can significantly decrease ROS production in the lungs and livers of female mice in TI + *E.coli* group (Figure 4D and 4E). Furthermore, DPI can inhibit the expression of serum inflammatory cytokines TNFα and IL-6 (Figure 4F). It is also verified DPI can inhibit mRNA expression of TNFα, IL1β and IL-6 in trained immunity J774 (Supplementary data, Figure SA). Together, these results demonstrated that ROS inhibition will weaken female sepsis resistant ability in trained immunity group. Also, ROS inhibition can decline immune intensity for fighting infections by suppress inflammatory cytokines production.

### Estradiol and trained immunity promote BMDM ROS level and enhance phagocytic ability related to RUBICON-mediated LAP

**Figure 5:**
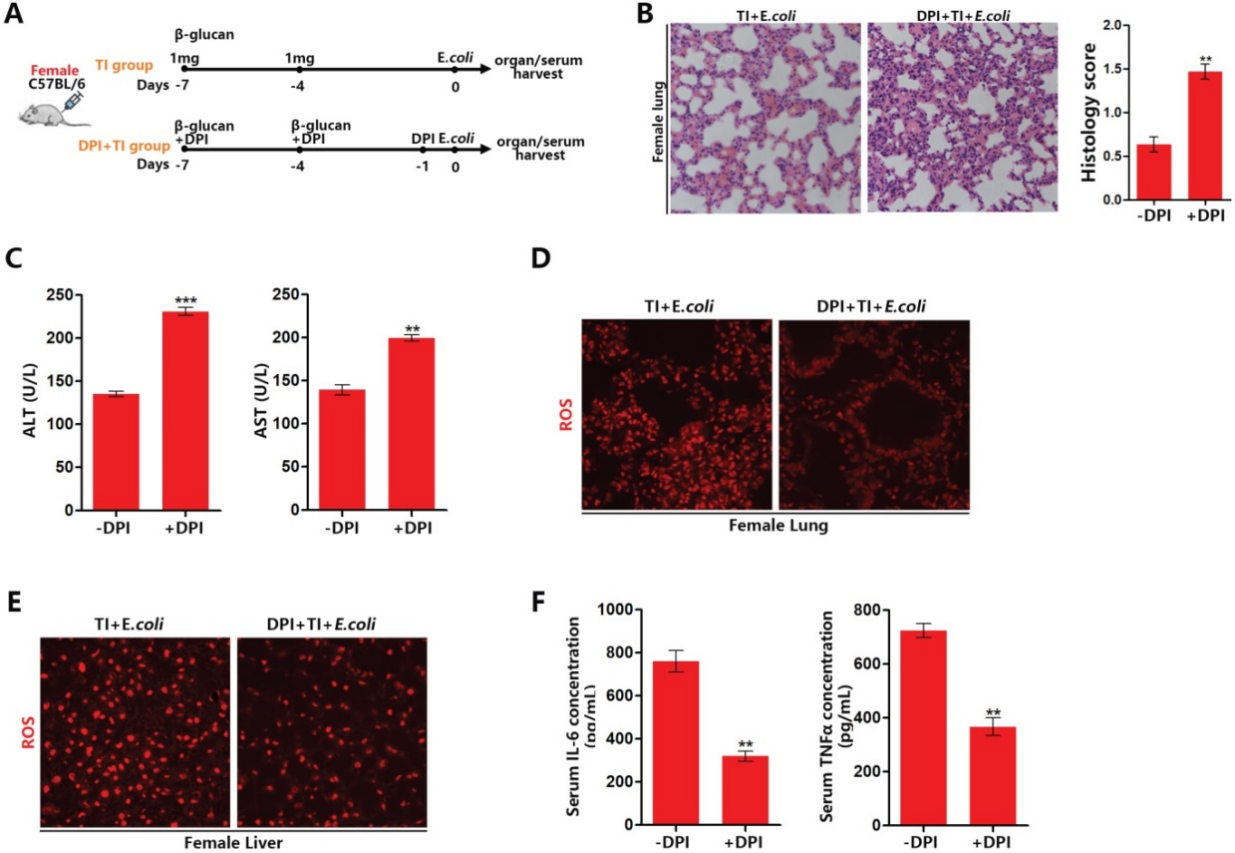
** Estradiol and trained immunity promote BMDM ROS level and enhance phagocytic ability related to RUBICON-mediated LAP.** (A) *In vitro* trained immunity model for BMDMs. (B) The mRNA levels of TNFα and IL-6 in BMDMs were detected by qPCR to determine the BMDMs TI model works. E_2_ and TI can promote BMDMs inflammatory effects. (C) E_2_ activated BMDMs Akt phosphorylation (hallmarks of trained immunity) by western blot. (D) Flowcytometric detection of total ROS of BMDMs in control, LPS, TI + LPS and E_2_ + TI + LPS groups. (E) The concentration of hydrogen peroxide and total amount of ROS of BMDMs in control, LPS, TI + LPS and E_2_ + TI + LPS groups. (F) Estradiol and trained immunity can upregulate expression level of LAP related proteins in BMDMs. (G) Immunofluorescence observation of the amount of *E.coli* particles engulfed by BMDMs at 15 min in particle, TI + particle and E_2_ + TI + particle groups. (H) Immunofluorescence observation found that estradiol and trained immunity promote the co-localization of RUBICON and p22^phox^ in BMDMs (n≥3/group). *p<0.05, **p<0.01 and ***p<0.001 when compared to control group. #*p<*0.05, ##*p<*0.01 and ###*p<*0.001 comparing LPS group and TI + LPS group. ^*p<*0.05, ^*p<*0.01 and ^^^*p<*0.001 comparing TI + LPS group and E_2_ + TI + LPS group.

When macrophages undergo autophagy, they produce autophagosomes to eliminate pathogens. However, autophagy can be divided into classic autophagy and non-canonical LAP. In addition, LAP can be initiated by TLRs on the cell membrane surface to identify pathogens. To confirm trained immunity can improve pathogen elimination ability by facilitating macrophage LAP, together with previous studies 33, we established trained immunity and sepsis model on BMDMs (Figure 5A). It is determined that estradiol and trained immunity can promote inflammatory cytokines production and the phosphorylation of Akt (Hallmark of trained immunity activation), indicating that BMDMs trained immunity model is effective (Figure 5B and 5C). Similarly, flow cytometry and fluorescence detection indicated that trained immunity can enhance intracellular ROS production in BMDMs (Figure 5D and 5E). Western blot showed that estradiol and trained immunity facilitated LAP-related proteins in BMDMs (Figure 5F). Immunofluorescence observation also proved that estradiol and trained immunity can stimulate BMDMs phagocytosis ability (Figure 5G). During the macrophage LAP, the combination of RUBICON with Beclin1 and p22^phox^ are very essential 34. Since RUBICON is an inhibitor of autophagy 35, it can stabilize the NOX2 structure to stably produce ROS by binding to p22^phox^ 21. Also, RUBICON can promote LAP. To assess BMDMs LAP is involved in phagocytic process, immunofluorescence observation showed that after estradiol and trained immunity stimulation, the co-localization of RUBICON and p22^phox^ can be promoted in BMDMs (Figure 5H). In addition, the co-localization of RUBICON and Beclin1 can also be promoted (Supplementary data, Figure SB). Since the autophagosomes in LAP are mainly produced by the accumulation of LC3B proteins on the surface of autophagosomes 36. Through immunofluorescence staining of LC3B, it is indicated that estradiol and trained immunity can increase the number of autophagosomes composed of LC3B (Supplementary data, Figure SC). Together, these results indicated that estradiol and trained immunity can stimulate BMDMs LAP by promoting the expression of LAP-related proteins and facilitate the co-localization of RUBICON and p22phox to eliminate pathogens.

### Estradiol promotes the RUBICON-mediated macrophage LAP through estrogen receptor α

**Figure 6:**
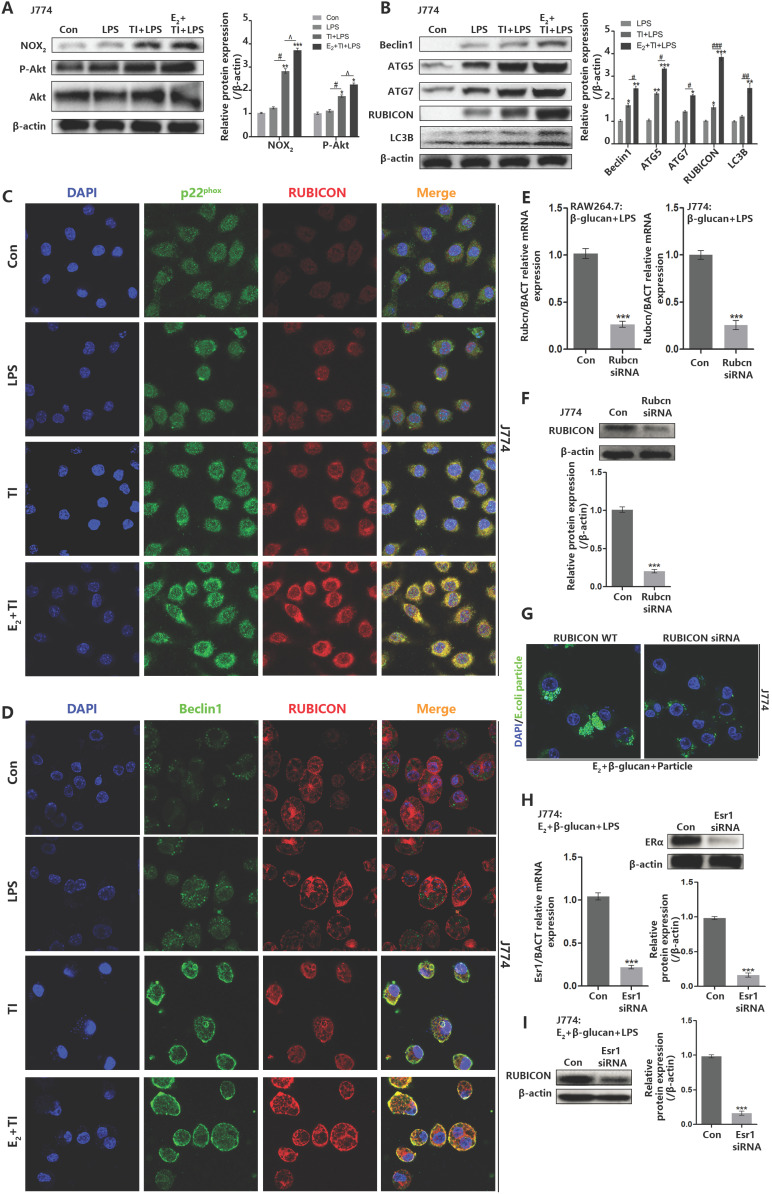
** Estradiol promotes the RUBICON-mediated macrophage LAP through estrogen receptor α.** (A) E_2_ activated J774 Akt phosphorylation (hallmarks of trained immunity) and NOX2 expression by western blot. (B) Estradiol and trained immunity can upregulate expression level of LAP related proteins in J774. (C) Immunofluorescence observation found that estradiol and trained immunity promote the co-localization of RUBICON and p22^phox^ in J774. (D) Immunofluorescence observation found that estradiol and trained immunity promote the co-localization of RUBICON and Beclin1 in J774. (E-F) qPCR and western blot verified Rubcn siRNA can knock down Rubicon expression in mRNA and protein level in J774. (G) Immunofluorescence observation found knockdown of RUBICON can weaken the phagocytic ability of J774 in E_2_ + TI + particle group. (H) qPCR and western blot verified Esr1 siRNA can knock down ERα expression in mRNA and protein level in J774. (I) Knockdown of ERα in J774 can inhibit RUBICON expression in E_2_ + TI + LPS group (n≥3/group). *p<0.05, **p<0.01 and ***p<0.001 when compared to control group or compared to LPS group (B). #*p<*0.05, ##*p<*0.01 and ###*p<*0.001 comparing LPS group and TI + LPS group or comparing TI + LPS and E_2_ + TI + LPS group (B). ^*p<*0.05 comparing TI + LPS and E_2_ + TI + LPS group.

Previous data suggested that trained immunity can promote macrophage LAP to phagocytose *E.coli*, and addition of E_2_ can further stimulate macrophage LAP. However, the promotion mechanism of estradiol needs further exploration. Western blot indicated that E_2_ and trained immunity can enhance Akt phosphorylation and NOX2 expression in J774 (Figure 6A) as well as LAP-related proteins (Figure 6B). Immunofluorescence observation found that E_2_ and trained immunity can enhance the co-localization of RUBICON and p22^phox^ in J774, and also promote membrane distribution of Beclin1 and its co-localization with RUBICON (Figure 6C and 6D). To assess the vital role of RUBICON for LAP, we used small RNA interference technology to knockdown RUBICON (Figure 6E and 6F). Knockdown of RUBICON can inhibit J774 LAP (Figure 6G). Estrogen receptors including ERα and ERβ can enter the nucleus to act as transcription factor by binding to E_2_. To investigate whether these two receptors are involved in regulating RUBICON expression and LAP, both receptors were knocked down by small RNA interference. After knockdown of ERα in J774, RUBICON expression can be reduced (Figure 6H and 6I), however knockdown of ERβ in J774 had no effect on RUBICON expression (Supplementary data, Figure SE and SF). We also verified that knockdown of ERα can suppress J774 LAP but not ERβ (Supplementary data, Figure SD). These data demonstrated that E_2_ can enhance the expression of RUBICON through ERα, thereby further promoting the enhancement effect of trained immunity on macrophage LAP.

## Discussion

Sepsis is a systemic inflammatory disease including systemic inflammatory response syndrome (SIRS) stage and compensatory anti-inflammatory response syndrome (CARS) stage during its initiation and development process 37. SIRS stage can be considered as early sepsis, in this stage, up-regulation of inflammatory response is beneficial to infection resistance. However, in the later CARS stage, immune system should relieve the inflammatory response in time 38. If immune cells failed to decrease cytokine production and eventually produce cytokine storm, will lead to organ damage even death 39. So sepsis can be considered as an autoimmune disease. The mechanism of trained immunity remains unclear. In this study, we found that trained immunity could promote macrophage LAP in early sepsis, in which the increased production of ROS and enhanced expression of RUBICON were necessary events. When fighting infection, pattern recognition receptors on the macrophage surface can not only up-regulate expression of inflammatory cytokines, but also enhance the LAP by recognizing danger signals. Since many patients with sepsis died because of excessive immunity caused by cytokine storm, it is of great scientific significance to explore how macrophages balance these two cell activities (cytokine production and LAP) against infection. Finding a method that only enhances the LAP process of macrophage instead of cytokine expression may be a feasible solution for the sepsis treatment in the future.

Estrogen is mainly synthesized in the ovary and was discovered as early as 1900s. Among them, estradiol is the most predominant estrogen circulating in the human body. The main estrogen receptors, including the nuclear receptors ERα, ERβ and the cell membrane receptor GPER1 40. The research on estrogen and its receptors mainly focuses on their role in breast cancer. When estrogen receptors bind to estradiol, they will be activated to enter the nucleus and become transcription factors. Also, they can activate downstream signaling pathways-NFκB signaling, for instance, to regulate gene expression 41. In addition, there exists complicated interactions among estrogen receptors. Although some studies have shown that estrogen can be used as a therapeutic target for sepsis 42, 43, the specific mechanism by which estradiol regulates inflammatory response and resists infection in sepsis through estrogen receptors needs further investigation.

The purpose of this paper was to elucidate the reasons for gender differences in autoimmune disease sepsis. There is limited research about how estradiol affects sepsis during trained immunity. Furthermore, as mentioned earlier in this article, gender dimorphisms in sepsis consist of many aspects, such as behavioral patterns, chromosomal differences with its X-chromosomal chimeric phenomenon and other hormone levels 44. The effects of estradiol and trained immunity on other types of immune cells are also of great scientific significance in context of autoimmune and infection diseases. Interfering with the signal pathway caused by estradiol and its receptor may be a solution to optimize sepsis treatment. Gender dimorphism in diseases is a very complicate and profound scientific problem, and the research in this area is very vital for precision medicine development, optimization of treatment for autoimmune diseases. Development of related molecular analogues or drugs for signaling pathway intervention needs further investigation to apply in clinical.

## Conclusion

In summary, the difference in hormone levels is a vital aspect of gender differences. As the most predominant estrogen, E_2_ participates in a bunch of life activities including immunity. We demonstrated that in early sepsis, β-glucan-induced trained immunity can upregulate NOX2 activity and expression to enhance ROS production in main organs. This indicated trained immunity can facilitate mice sepsis resistant ability. In the meanwhile, we also found females in trained immunity survived better than males. By establishing trained immunity and sepsis model in OVX mice, the sepsis resistant ability significantly decreased which suggested that estradiol can promote trained immunity. In addition, by i.p. injecting NADPH oxidase inhibitor DPI into female mice with trained immunity and sepsis model, we found prevent ROS production can also reduce sepsis resistant effect. Because phagocytosis is a significant way for macrophage to eliminate pathogens, and ROS is closely related to LAP. In order to further clarify the effect of E_2_ and trained immunity on LAP, we established trained immunity and sepsis model on macrophage cell lines and BMDMs *in vitro*. The results indicated that trained immunity not only can produce more ROS by up-regulating NOX2 activity, but also increase RUBICON expression, to jointly promote macrophage LAP. E_2_ can further facilitate the promotion effect mentioned above. In a word, the gender dimorphism in early sepsis resistance comes from macrophage LAP enhancement caused by estradiol and trained immunity. These results may provide a possible reference way to optimize the clinical treatment of autoimmune diseases and sepsis.

## Materials and Methods

### Reagents and antibody

17β-Estradiol (50-28-2), β-glucan (G-59303) from yeast were purchased from XiEnSi biotechnology company (Tianjin, China). DPI was purchased from Selleck (Shanghai, China). M-CSF (#CB34) and IFN-γ (#C746) for BMDMs were purchased from Novoprotein (Shanghai, China). LPS was purchased from Sigma (St. Louis, MO, USA). Antibody of NOX2 (19013-1-AP), Beclin1 (66665-1-Ig) and Rubicon (21444-1-AP) were purchased form proteintech (Wuhan, China). Estrogen receptor β (ab288) was purchased from Abcam (Shanghai, China). P-Akt (Ser235/236, #4060), Akt (#4691), Estrogen receptor α (13258s), β-actin (#3700), LC3B (#83506), ATG5 (#12994) and ATG7 (#8558) were purchased from Cell Signaling (Boston, MO, USA).

### Cell culture

Macrophage cell lines RAW264.7 and J774 were obtained from the Type Culture Collection of the Chinese Academy of Sciences, Shanghai, China. Cells were cultured in phenol red-free DMEM with 1% penicillin-streptomycin solution (100 U/mL penicillin and 100 μg/mL streptomycin) and 10% FBS (Fetal bovine serum) at 37 °C in an atmosphere of 95% air and 5% CO_2_. Cells were used within 5 passages at maximum, and were seeded onto different types of plates for further experiments when cell density reached approximately 70%.

RAW264.7 and J774 trained immunity model was established *in vitro* by using 5 μg/mL β-glucan treatment for 24 h. Thereafter, cells were washed with PBS and rested in culture medium for 5 days. With second stimulation with 100 ng/mL LPS, cells were harvested for later experiments.

### BMDM isolation and culture

Mice were sacrificed by cervical dislocation and sterilized by soaking in 75% ethanol. After the legs were dissected, bone marrow was extracted from tibia and femur bones by using a 25-gauge needle and a 1 mL syringe filled with PBS following removal of surrounding muscle tissues. Blow the bone marrow gently and spread it through a 70 μm cell strainer. The cell suspension was centrifuged at 300 g for 5 min at room temperature. Then, cells were cultured in phenol red-free DMEM with 10% ultra-low endotoxin FBS and 20 ng/mL M-CSF. After changing the fresh medium on the third and fifth day, we can get BMDMs.

### BMDM trained immunity model

After obtaining mature BMDMs, BMDMs were challenged with 100 μg/mL β-glucan for 24 h. Then cells were washed by PBS and rested for 3 days in culture medium. On day 4, BMDMs were washed again and stimulated with 25 ng/mL IFN-γ for 24 h. Then, a final wash was performed where cells were primed with 1 μg/mL LPS. Experiments were based on the same model but with different numbers and densities of BMDMs.

### Mice

Male and Female C57BL/B mice were purchased from Model Animal Research Center of Nanjing University. 10-week-old mice were used in the model of trained immunity and sepsis. Female mice were anesthetized with 4% chloral hydrate and then underwent ovariectomy (OVX) at six weeks of age. When they grow up to 10 weeks old, the same trained immunity and sepsis model was established. All animal procedures were performed in accordance with guidelines of the US NIH with Specific Pathogen Free conditions. Soy-free standard rodent chow and water were provided *ad libitum* to minimize the effect on mice estrogen levels.

### *In vivo* trained immunity and sepsis model

Mice were trained with two intraperitoneal (i.p.) injections of 1mg β-glucan particles on day -7 and -4. Control group was used sterile PBS. On day 0, mice were challenged with E. *coli*. The lung, liver, kidney and serum were harvest at proper time point after E. *coli* treatment.

### *E.coli* stain preparations

The E.*coli* 15597 stains were obtained from ATCC, collected, and identified by the Medical Laboratory Center of Zhongda Hospital in Nanjing, Jiangsu, China. Stored at -80 °C. Bacterial strains were prepared in LB medium.

### siRNA transfection

siRNA was transfected according to the product instructions (Ruibo company, China). The concentration of siRNA used in the study was 50nM. The ERα siRNA target sequence is TGCACATTGAAGATGCTGA. The ERβ siRNA target sequence is GGTCCTGTGAAGGATGTAA, The Rubicon siRNA target sequence is CCCACTCGGACACCAACAT. The non-coding sequence for negative control was a random sequence with no biological effects.

### Quantitative PCR

Total RNA was extracted from cells by using TRIzol reagent, and then reverse transcriptions were performed according to the manufacturer's instructions in a 20μL mixture with 1 μg of total RNA (Vazyme company, China). The oligonucleotide primers used for PCR amplification are listed in Table 1, PCR amplification consisted of 30 cycles of denaturation at 95 °C for 2 min, annealing at 60 °C for 45 s, and extension at 72 °C for 2 min. All reactions were run in triplicate. The gene expression level were normalized to β-actin.

### Western blotting

The protein samples were obtained from RIPA lysis buffer treated cells. Cell lysates were put on ice for 15 min and then centrifuged at 12000 rpm for 10 min at 4 °C. Subsequently, 30 μg protein per lane was separated on 10% or 15% polyacrylamide gels and transferred onto polyvinylidene difluroride membranes (Millipore, Billerica, MA, USA). Membranes were blocked with 5% bovine serum albumin (BSA) in Tris-buffered saline containing 0.1% Tween 20, and then the membranes were incubated with specific antibodies in proper concentration at 4℃ overnight. The values were normalized to the β-actin intensity levels.

### H&E staining

The fresh lung tissues were fixed in 4% paraformaldehyde (PFA). Then, the samples were gradually dehydrated and embedded in paraffin. After that, the samples were cut into 3 μm sections and stained with hematoxylin and eosin for further light microscopy observation. Scores were evaluated by a pathologist based on the lung tissue integrity, alveolar integrity, and mononuclear infiltration. (0=none; 1=mild; 2=moderate; 3=severe).

### Immunohistochemistry

The fresh lung tissues were fixed in 4% paraformaldehyde (PFA). Then, the samples were gradually dehydrated and embedded in paraffin. Organ sections were cut into 5μm in thickness and then deparaffinized and incubated in citrate buffer at 95 °C for 40 min for antigen retrieval and then incubated overnight at 4 °C with NOX_2_ primary antibody (1:100 dilution, proteintech, 19013-1-AP). After three washes, tissue slices were incubated with biotinylated anti-mouse IgG (1:200 dilution, Vector Laboratories, CA, USA) for 1 hour at RT and then washed three times, after which streptavidin-horseradish peroxidase conjugates (Vector Laboratories, CA, USA) were added and the slices incubated for 45 min. After three washes with PBS, DAB solution (Vector Laboratories, CA, USA) was added and the slides were counterstained with hematoxylin.

### ROS staining in tissue frozen section

Fresh tissues were embedded in OCT (#4583, Sakura, Atlanta, USA) and cut into 10 μm thick slices with a frozen microtome. The autofluorescence of the tissue slices were removed by Servicebio Autofluorescence quencher (#G1221, Wuhan, China), slices then were washed with PBS and conducted ROS staining with 1:200 ROS stain (#G1045, Servicebio, Wuhan, China). After 3 washes with PBS, tissue slices were sealed with neutral resin. Observe and take pictures with microscope at 590 nm wavelength.

### Macrophage phagocytosis assay

To evaluate macrophage phagocytosis intensity, pHrodo Green E. coli BioParticles Conjugate was purchased (P35366) from ThermoFisher (Shanghai, China). The reagent was used according to manufacturer's instruction. Cells are harvested for following flow cytometry analysis and immunofluorescence.

### ROS detection assay

ROS detection kit (S0033) was purchased from Beyotime (Shanghai, China). 10 μM (final concentration) DCFH-DA was added to culturing macrophages according to manufacturer's instruction, cells can be harvested for flow cytometry analysis or absorbance detection at 488 nm.

### H_2_O_2_ detection assay

Hydrogen Peroxide assay kit (S0038) was purchased from Beyotime (Shanghai, China). The concentration of H_2_O_2_ in cells was detected according to manufacturer's instruction.

### Flow cytometry analysis

Macrophages were filtered through a 70 μM cell strainer and then washed with complete RPMI medium to generate single-cell suspensions. Macrophages tested for phagocytosis activity and ROS production were detected by a FACS Calibur flow cytometer (BD Bioscience) and data were analyzed using FlowJo software (TreeStar, Ashland, OR). All flow cytometry data are stained with only one dye (DCFH-DA), the gating strategy is based on the blank control.

### Immunofluorescence

Cultured cells were seeded on glass coverslips in six-well plates. After three PBS washes, the samples were fixed for 15 min at RT with 4% paraformaldehyde. Fixed cells were rinsed with PBS and then incubated for 10 min at 4 °C with 0.2% Triton X-100 and 0.2% BSA in PBS. Following permeabilization nonspecific binding in the cells was blocked by 5% BSA in PBS for 1h at RT. Cell samples were incubated with anti-p22-phox, anti-Beclin1, anti-Rubicon and anti-LC3B primary antibodies at 1:200 dilution for 2h at RT. Samples were further incubated with Alexa Fluor-488-conjugated and Alexa 647-conjugated secondary antibody at a 1:350 dilution for 1.5h in the dark. After washed with PBS, the nuclei were stained by DAPI. Slides were visualized using a Nikon Eclipse Ti-U fluorescence microscope equipped with a digital camera (DS-Ril, Nikon).

### ELISA

The protein concentration of IL-6, TNFα and estrogen in cell supernatant or mouse serum were detected using the corresponding mouse enzyme-linked immunosorbent assay (ELISA) kit according to the manufacturer's instruction (Biolegend, China).

### Kidney burden

The kidney E. coli burden at indicated time points was measured by plating organ homogenates obtained mechanically over 70 μm cell strainers (BD Biosciences) following slicing the tissue, in serial dilutions on LB agar plates; colony- forming units (CFUs) were counted after growth at 37 °C for 24 h, and data are shown as CFUs in one total kidney.

### Statistical analysis

The statistical analysis was performed using Prism (Prism 5 for Windows, Graphpad Software Inc., USA). Unless specified, statistical significance for comparison between two sample groups with a normal distribution (Shapiro-Wilk test for normality) was determined using two-tailed paired or unpaired Student's t test. Data from more than two groups were analyzed by one-way analysis of variance (ANOVA) of the differences within each treatment, and Tukey's *post hoc* test was used. Differences were considered significant at *p<*0.05 as indicated. Except when specified, only significant differences are shown. As indicated in figure legends, either a representative experiment or a pool is shown, and the number of repetitions of each experiment and number of experimental units (either cultures or mice) is indicated. The results are presented as the means *±* standard error (SEM).

## Highlights

- E_2_ facilitates β-glucan-induced trained immunity to increase female sepsis resistance better than males;
- β-glucan-induced trained immunity promotes NOX2 expression and ROS production to enhance macrophage LC3B-associated phagocytosis (LAP);
- E_2_ can further promote LAP of trained macrophages through ERα;
- The different E_2_ levels *in vivo* may be one of the reasons why men and women have different tolerance to sepsis.

## Supplementary Material

Supplementary figures and tables.

## Figures and Tables

**Table 1:** Primer sequences

Gene	Sense (5'-3')	Anti-sense (5'-3')
Nox1	GGTTGGGGCTGAACATTTTTC	TCGACACACAGGAATCAGGAT
Nox2 (Cybb/gp91-phox)	TGTGGTTGGGGCTGAATGTC	CTGAGAAAGGAGAGCAGATTTCG
Nox3	CAACGCACAGGCTCAAATGG	CACTCTCGTTCAGAATCCAGC
Nox4	GAAGGGGTTAAACACCTCTGC	ATGCTCTGCTTAAACACAATCCT
Duox1	AAAACACCAGGAACGGATTGT	AGAAGACATTGGGCTGTAGGG
Duox2	AAGTTCAAGCAGTACAAGCGAT	TAGGCACGGTCTGCAAACAG
TNFα	CAGCAAGGGACAGCAGAGG	AGTATGTGAGAGGAAGAGAACC
IL6	TAGTCCTTCCTACCCCAATTTCC	TTGGTCCTTAGCCACTCCTTC
IL1β	GCAACTGTTCCTGAACTCAACT	ATCTTTTGGGGTCCGTCAACT
p47-phox	ACACCTTCATTCGCCATATTGC	TCGGTGAATTTTCTGTAGACCAC
Sod1	AACCAGTTGTGTTGTCAGGAC	CCACCATGTTTCTTAGAGTGAGG
Gpx1	AGTCCACCGTGTATGCCTTCT	GAGACGCGACATTCTCAATGA
Gpx2	GCCTCAAGTATGTCCGACCTG	GGAGAACGGGTCATCATAAGGG
Catalase	AGCGACCAGATGAAGCAGTG	TCCGCTCTCTGTCAAAGTGTG
Rubcn	CAGGGTGTAGTGCATGGTTCT	CCGCCAAGATCCATTCCCG
Esr1	CCTCCCGCCTTCTACAGGT	CACACGGCACAGTAGCGAG
Esr2	CTGTGCCTCTTCTCACAAGGA	TGCTCCAAGGGTAGGATGGAC
BACT	GGCTGTATTCCCCTCCATCG	CCAGTTGGTAACAATGCCATGT

## Abbreviations

E_2_: 17-β-estradiol
TI: trained immunity
LPS: lipopolysaccharide
NOX2: NADPH oxidase-2
PBS: phosphate buffer saline
AST: aspartate aminotransferase
ALT: alanine aminotransferase
TNFα: tumor necrosis factor α
IL-6: interleukin-6
IL-1β: interleukin-1β
SOD1: superoxide dismutase 1
GPX1: glutathione peroxidase 1
GPX2: glutathione peroxidase 2
Esr1: estrogen receptor 1
M-CSF: Macrophage Colony-stimulating factor
IFN-γ: interferon-γ
Rubicon: RUN domain and cysteine-rich domain containing, Beclin 1-interacting protein
