# Supplementary material for: 17β-Estradiol promotes LC3B-associated phagocytosis in trained immunity of female mice against sepsis
Source: Int J Biol Sci. 2021 Jan 1;17(2):460–74. doi: 10.7150/ijbs.53050 (PMC7893586; doi:10.7150/ijbs.53050)
Supplement: Supplementary file 1 — Supplementary figures and tables. [file ijbsv17p0460s1.pdf]

Supplemental Data

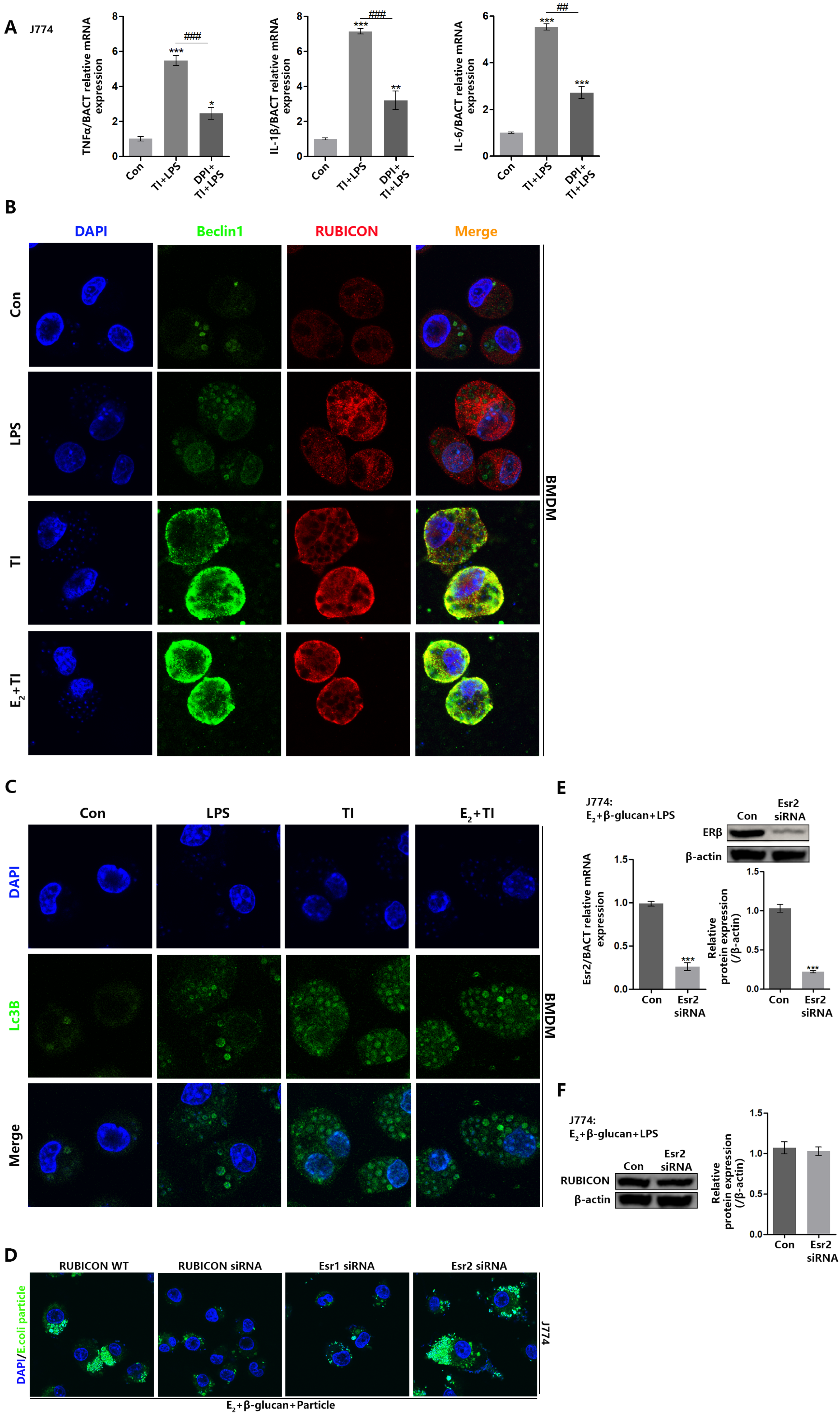

Supplemental data:

Figure SA: The addition of NOX2 inhibitor DPI to trained immunity J774 suppresses inflammatory cytokines expression. The mRNA levels of TNFα, IL-1β and IL-6 in J774 were detected by qPCR to determine the effect of DPI on inflammatory cytokines in TI + LPS group (n≥3/group). *p<0.05, **p<0.01 and ***p<0.001 when compared to control group. ##p<0.01 and ###p<0.001 comparing TI + LPS and DPI + TI + LPS group.

Figure SB: Estradiol and trained immunity facilitate the co-localization of RUBICON and Beclin1 in BMDMs. Immunofluorescence observation found that estradiol and trained immunity promote the co-localization of RUBICON and Beclin1 in BMDMs (n≥3/group).

Figure SC: Estradiol and trained immunity increased the number of LC3B formed autophagosomes in BMDMs LAP (n≥3/group).

Figure SD: Knockdown of estrogen receptor α in J774 reduced phagocytic ability of J774 in E_2 + TI + particle group, however knockdown of estrogen receptor β in J774 had no effect on phagocytic ability of J774 in E2 + TI + particle group (n≥3/group).

Figure SE: qPCR and western blot verified Esr2 siRNA can knock down ERβ expression in mRNA and protein level in J774.

Figure SF: Knockdown of ERβ in J774 had no effect on RUBICON expression in E_2 + TI + LPS group (n≥3/group).
